# Supplementary figures and images for: The effect of growth rate on otolith-based discrimination of cod (Gadus morhua) ecotypes
Source: PLoS One. 2021 Sep 29;16(9):e0247630. doi: 10.1371/journal.pone.0247630 (PMC8480848; doi:10.1371/journal.pone.0247630)

Otolith image:


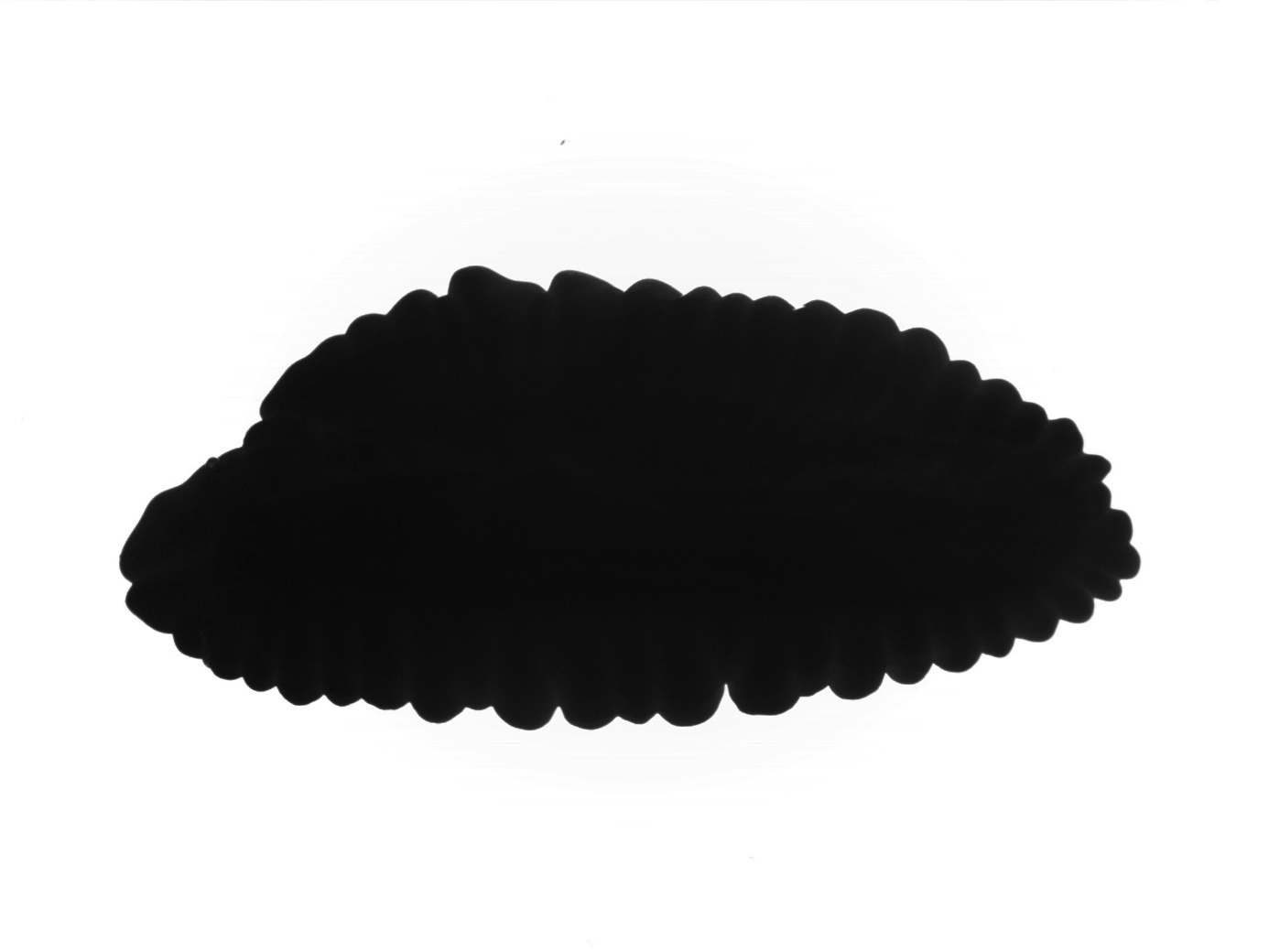


Fourier reconstruction:


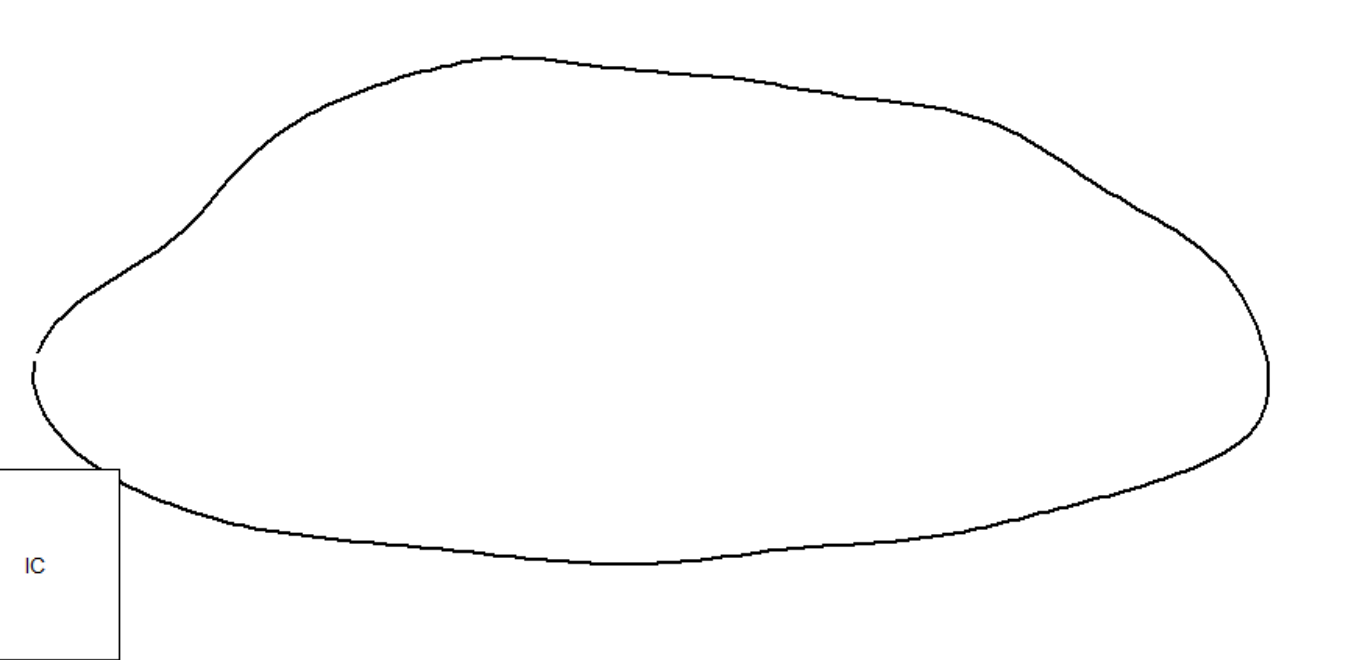


Wavelet reconstruction:


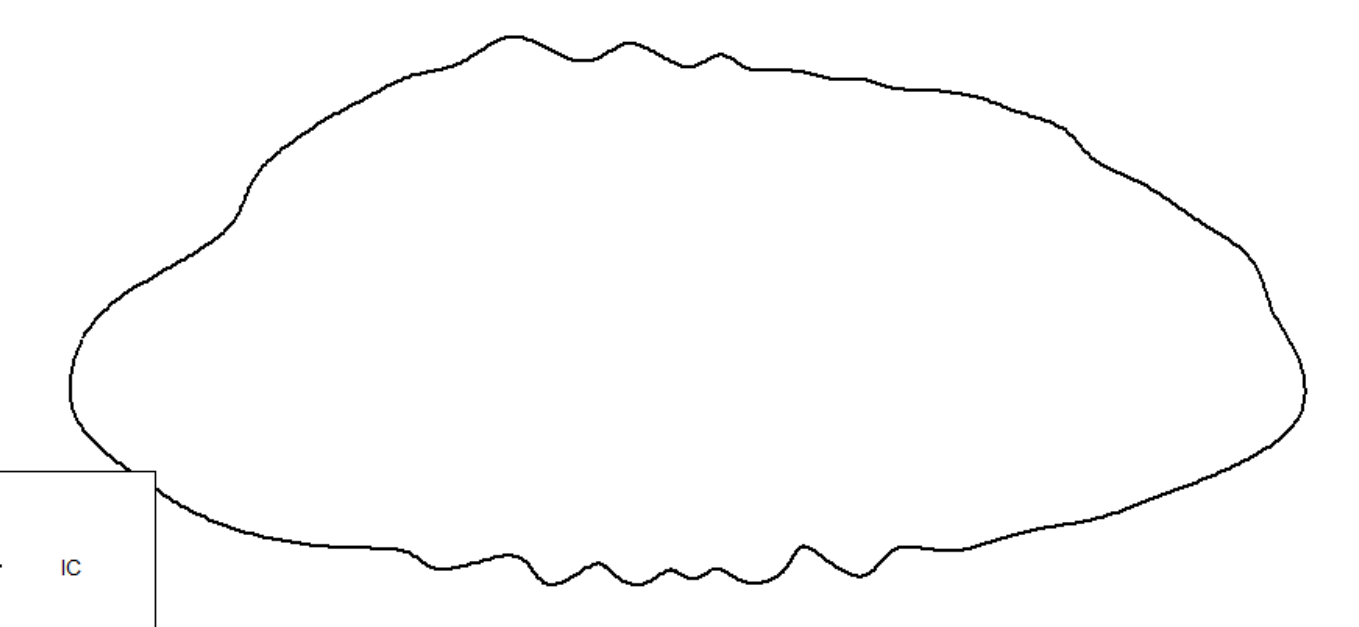

Supplement: S1 Fig — (DOCX) [file pone.0247630.s001.docx]
